# Exosome-Based Liquid Biopsy Approaches in Bone and Soft Tissue Sarcomas: Review of the Literature, Prospectives, and Hopes for Clinical Application

**DOI:** 10.3390/ijms24065159

**Published:** 2023-03-08

**Authors:** Chiara Agnoletto, Ymera Pignochino, Chiara Caruso, Cecilia Garofalo

**Affiliations:** 1 Veneto Institute of Oncology IOV—IRCCS, 35128 Padua, Italy; 2 Department of Clinical and Biological Sciences, University of Torino, 10043 Torino, Italy; 3 Candiolo Cancer Instute, FPO-IRCCS, 10060 Torino, Italy; 4 Advanced Translational Research Laboratory, Immunology and Molecular Oncology Diagnostic Unit, Veneto Institute of Oncology IOV—IRCCS, 35127 Padua, Italy

**Keywords:** sarcoma, exosomes, liquid biopsy, preclinical and clinical practice

## Abstract

The knowledge of exosome impact on sarcoma development and progression has been implemented in preclinical studies thanks to technological advances in exosome isolation. Moreover, the clinical relevance of liquid biopsy is well established in early diagnosis, prognosis prediction, tumor burden assessment, therapeutic responsiveness, and recurrence monitoring of tumors. In this review, we aimed to comprehensively summarize the existing literature pointing out the clinical relevance of detecting exosomes in liquid biopsy from sarcoma patients. Presently, the clinical utility of liquid biopsy based on exosomes in patients affected by sarcoma is under debate. The present manuscript collects evidence on the clinical impact of exosome detection in circulation of sarcoma patients. The majority of these data are not conclusive and the relevance of liquid biopsy-based approaches in some types of sarcoma is still insufficient. Nevertheless, the utility of circulating exosomes in precision medicine clearly emerged and further validation in larger and homogeneous cohorts of sarcoma patients is clearly needed, requiring collaborative projects between clinicians and translational researchers for these rare cancers.

## 1. Introduction

Exosomes are extracellular vesicles of 30–100 nm in diameter originated through the inward budding of multivesicular bodies and released at the cellular plasma membrane into the extracellular compartment [1,2,3]. Upon release, they circulate in blood vessels and accumulate in body fluids, with relative stability [1,4,5,6]. Physiologically, circulating exosomes have heterogeneous origin (e.g., from platelets, lymphocytes, dendritic cells, and other immune cells) and constitute 80–90% of serum/plasma extracellular vesicles [7], sharing certain characteristics, including shape, size, density, and composition [1]. It was demonstrated that exosomes are generated by most cell types at low levels [8], while they are actively released by neoplastic cells, having a role in tumor transformation and progression [1,9,10,11]. Of note, exosomes present and contain specific arrays of lipids, RNAs (e.g., mRNA, miRNA, lncRNA), and biologically active proteins, which constitute tissue and disease-distinct functional fingerprints and reflect ongoing cellular activities. In accordance, high throughput proteomic studies of exosomes isolated from diverse cells have identified and systematically mapped thousands of vesicular proteins [12,13,14], demonstrating the existence of both specific tumor-type molecules and a common set of components serving as molecular signatures of their cell of origin [1,15,16]. Several reports in the literature confirm the major role of exosomes in cellular communication in tumors, since once captured by recipient cells, they activate function-altering programs through instructions conveyed by a specific array of molecules [17,18,19]. Indeed, exosomes influence stromal, endothelial, inflammatory, and immune cells’ functions toward a pro-tumorigenic phenotype [8]; for instance, they promote tumor growth and invasion [20], they induce neovascularization and extracellular matrix remodeling [21,22], prepare pre-metastatic niches [20,23,24], facilitate anti-tumor innate and adaptive immune responses [4,25,26,27,28], and modulate drug resistance [29]. Consistently, quantitative changes in tumor exosomes and their cargoes have been detected through longitudinal analysis of clinical samples [30], and they obviously represent potential novel diagnostics or therapeutic tools of great interest in a clinical setting.

Active components include miRNAs, small, non-coding RNA molecules consisting of 20–22 nucleotides [31,32]. Expression of miRNAs has been extensively documented to be frequently altered in several human tumors, and they exert a crucial role in tumor initiation, progression, and metastasis, acting both as oncogene or tumor suppressors [33,34,35,36]. Recent evidences confirmed that tumor cells actively secrete miRNAs into the circulation, protected by exosomes or argonaute 2 [3,33,37,38]. miRNA expression profiles may be predictively associated with different tumor types at different stages, and a lot of studies have assessed the potential use of serum or plasma miRNAs as novel diagnostic biomarkers and in monitoring the effects of therapeutic interventions in several cancers [34,35,37]. Moreover, sequence motifs controlling the localization of miRNAs into exosomes have been recently identified and the active sorting mechanism of miRNAs in these vesicles was elucidated [39].

**Figure 1:**
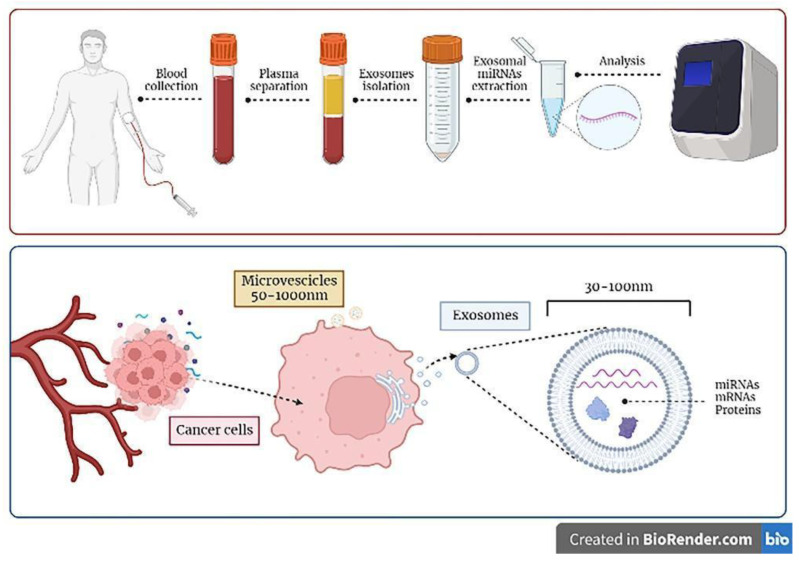
Principles of exosomes-based liquid biopsy. Exosomes can be detected in the serum or plasma of patients diagnosed with sarcoma by collecting blood samples of patients; exosomes can be selectively isolated and further characterized in their content (e.g., miRNAs molecules), which can be analyzed with the most advanced technologies (i.e., next generation sequencing analysis) in order to be informative. As depicted in the lower panel of the figure, cancer cells constitutively release exosomes and microvescicles in the extracellular medium, which then enter the circulation. Exosomes derive by the inward budding of cellular multivesicular bodies and contain informative molecules, e.g., miRNA, mRNAs, and proteins, that could be transferred to recipient cells.

Liquid biopsy based on tumor-derived exosomes represents a promising method for tumor monitoring or prognostic prediction [40,41] Numerous pieces of evidence have demonstrated the utility of circulating exosomes, providing shared and tumor-specific genomic and proteomic signatures [42] (see Figure 1), as proven in glioblastoma [10,43,44], melanoma [23,43], prostate [45,46], ovarian [47,48], lung carcinoma [49], gastrointestinal stromal tumor [50], pancreatic cancer [51], acute myeloid leukaemia [52], breast [53,54], and colorectal cancers [55]. Altogether, these studies support the use of patient-derived exosomes as non-invasive tools for the early diagnosis and monitoring of tumor aggressiveness and chemotherapeutic sensitivity. Moreover, recent reviews specifically discussed the role of exosomes in sarcomas [56,57,58]. However, they have not been translated into standard clinical practice yet, and more studies are needed to confirm the reproducibility in the detection of exosomes [40,41,50].

Novel targets for liquid biopsy include circulating tumor cells (CTCs) and nucleic acids such as circulating tumor DNA (ctDNA). Although CTCs can be isolated from blood, they are extremely rare cells, and several issues with the sampling of captured cells further limit clinical applicability [59,60,61]. ctDNA has similar limitations for early stage cancers, despite recent advances in technologies for ctDNA detection [61]. In contrast, exosomes-based approaches display potential superior sensitivity than ctDNA/CTCs, even in early stage cancer [40,41,62,63]. Moreover, exosome isolation methods represent relatively easy procedures with contained costs [64] compared to methods targeting ctDNA or CTCs. However, the exosome isolation for biomarker identification as liquid biopsies has not been utilized in large clinical trials so far. In fact, the discrimination of tumor exosomes from extracellular vesicles (EVs) of other pathological or physiological origin, and the very low concentration of tumor markers as scarce traces make this application very hard so far. Nevertheless, from preliminary studies presenting improved sensitivity of detecting methods, it has clearly emerged that tumor exosomes are potentially a powerful source of biomarkers with utility in diagnosis, prognosis prediction, tumor burden assessment, therapeutic responsiveness evaluation, and recurrence monitoring, and could represent advancement in precision medicine.

In the present manuscript, we discuss the current relevance of usage of exosomes in liquid biopsy in sarcoma patients, focusing on clinical data highlighting their identification and monitoring in serum for precision medicine applications.

## 2. Clinical Utility of Exosomes Detection in Liquid Biopsy

In this section, the most recent reports in the literature pointing to exosomes as liquid biopsy from patients diagnosed with bone and soft tissue sarcomas (BSTSs) are presented. In particular, evidences on the clinical impact of exosomes detection have been summarized. The most relevant data are briefly reported in Table 1, and described in detail in the next paragraphs.

BSTS are heterogeneous tumors of mesenchymal origin with more than 100 histological subtypes [65], and each BSTS histotype presents a specific nucleic acid or protein profile, which allows molecular diagnosis of sarcoma [66,67].

The Ewing Sarcoma Family of Tumors (ESFT) constitutes a group of primary pediatric osseous and soft tissue tumors, and consists of poorly differentiated small round blue cells with minimal stroma [68]. ESFT includes Ewing sarcoma of the bone, extraosseous Ewing sarcoma (ES), and peripheral primitive neuroectodermal tumors (pPNET), and most ESFT patients present micrometastatic disease at diagnosis [69]. Over the past few decades, the diagnosis of ES has become more accurate due to detection of relevant hallmarks, including CD99/MIC2 in immunohistochemistry and the oncogenic fusions of the Ewing sarcoma RNA binding protein 1 gene (*EWSR1*) [70,71]. ES is characterized by highly recurrent translocations involving ETS transcription factors, with EWS-FLI1 and EWS-ERG being the most common [72,73]. EWS-FLI1 induces a gene expression signature that ultimately dictates the malignant phenotype of ES [74]. Diagnostic approaches in a routine setting rely on invasive biopsy sampling of tumor tissue [75], and no liquid-based assays in clinical practice for diagnosing ESFT are available, evaluating minimal residual disease and onset of rescue therapies [76].

Microarray analysis of ES exosomes revealed that they share a common transcriptional signature potentially involved in intercellular communication, i.e., G-protein-coupled signaling, neurotransmitter signaling, and stemness [77]. Of note, the top five markers (NR0B1, NKX2.2, STEAP1, LIPI, and EWS-FLI1) were not detectable in the peripheral blood of healthy donors [77]. EWSR1-FLI1 induces the expression of the Polycomb histone methyltransferase EZH2 in ES cells in vivo and human mesenchymal stem cells (MSC) in vitro [78], which participates in the maintenance of cell pluripotency [79] and oncogenic transformation, and correlates with poor prognosis [78]. Additionally, EZH2 mRNA into exosomes has been detected in plasma of ES type 1 patients, and not healthy donors or patients with other types of sarcoma tumors [80]. Thus, assaying circulating exosomes can help in diagnosis of ESFT and potentially in predicting response to therapy and recurrence [71].

Liquid-based immuno-enrichment for ESFT-specific exosomes has been performed using CD99 and NGFR, and the EWS-ETS fusion transcript has been detected with high specificity from as little as 250 μL of plasma samples of 10 metastatic and localized pediatric patients, with a significant diagnostic power (AUC = 0.92, *p* = 0.001 for sEV numeration) [71]. CD99 prevents cell differentiation in order to maintain the proliferative as well as the metastatic capabilities of tumor cells [81,82]. NGFR has been implicated in the paracrine growth regulation of a number of neuronal and non-neuronal tumor types, and altered expression has been reported in sarcoma [71,83,84]. Immunocapture of ESFT exosomes may significantly increase the sensitivity in the detection of EWS-FLI1 Types I, II, and III and EWS-ERG fusion transcripts present with low frequency, circumventing the sequencing of patient-specific DNA breakpoints in tumor tissue [85].

Additionally, the quantitative measurement of EWS-FLI1 mRNA copy numbers in pPNET-derived exosomes represents an effective biomarker signature with respect to total cell RNA content, increasing the sensitivity for MRD identification during therapy and post-therapy [86]. Analogously, ESFT patients have been proven to present a higher content of exosomal miRNAs, with an average of 275 exo-miRNAs identified in ESFT pediatric patients and <100 exo-miRNAs identified in pediatric non-cancer, rhabdomyosarcoma, and OS samples [87]. Of note, Pearson’s clustering of 46 exo-miRNAs correctly identified 80% (4 of 5) of pathology-confirmed ESFT patients, with respect to healthy controls and 75% (3/4) of the non-ESFT sarcoma samples [87]. Importantly, RNAseq analysis of tumor tissue from the one outlier revealed a previously uncharacterized EWS-FLI1 translocation [87].

Last, exosomes content includes also molecules that induce inflammatory responses and immunosuppression, which are crucial determinants in ES [88,89], and are associated with systemic inflammation and poor prognosis [90]. Levels of LINE, SINE, and ERV retroelements and locus-specific pericentromeric chromatin-derived transcripts (L1, HERV-K, HSAT2, and ACRO1) in plasma exosomes correlate with inflammation [91] and metastatic progression [92]. Upon capture into stromal fibroblasts and immune cells, they promote the expansion of myeloid-derived suppressive cells (MDSCs), and tolerogenic and exhausted CD8+ T-cells; in addition, repeated RNAs were transmitted in recipient cell exosomes, with co-occurrence of inflammation and immunosuppression, eventually compromising antitumor immunity [92].

Myxofibrosarcoma (MFS) comprises a spectrum of malignant fibroblastic lesions with variably myxoid stroma, pleomorphism, and a distinct vascular pattern [92]. Infiltrative growth is a major cause of frequent recurrence, distant metastasis, and tumor-related death [93,94]. At present, effective biomarkers for monitoring tumor recurrence are still lacking. Recently, the profiling of circulating miRNAs in patients with MFS has been described, confirming their functional role in local MFS aggressiveness [95]. Dissimilar deregulation patterns were observed between intracellular and extracellular miRNAs [95], as reported in OS [33] and synovial sarcoma [96]. miRNA profiling identified four upregulated miRNAs in MFS patient sera, namely miR-642a, miR-1260b, miR-4286, miR-4313, and serum miR-1260b levels were closely correlated with clinical status and tumor dynamics [95]. Indeed, miR-1260b mediates cellular infiltration in vitro by downmodulating the expression of the adhesion molecule PCDH9 in adjacent normal fibroblasts, possibly inhibiting adhesion between tumor and normal mesenchymal cells in the microenvironment [95]. Of clinical relevance, the serum miR-1260b levels significantly decreased postoperatively in all tested patients, acting as a biomarker for non-invasive tumor monitoring of this highly aggressive sarcoma [95].

Osteosarcoma (OS) is the most frequent primary tumor of bone [97]. The presence of metastasis at diagnosis in 10–20% of all patients predicts a poor clinical outcome, while 30–40% of patients without metastasis at diagnosis will relapse independently of therapy [98]. Pulmonary metastases represent the main cause of death [98,99]. However, the majority of patients at diagnosis have undetectable micrometastases, with a 5-year survival rate inferior at 20%, while non-responsive to aggressive chemotherapy [100]. To date, detection of metastasis at diagnosis and histopathologic response to neoadjuvant chemotherapy remain, in clinical settings, the most effective predictors of outcome [101]. Additionally, current clinical markers still have the better prognostic significance, due to the heterogeneous nature of these tumors [102].

Exosomes from OS cells have been proven to exert an important role in tumor progression and metastasis [103], and can be utilized as a biomarker to monitor tumor progression [104,105,106]. However, isolation of exosomes from serum of OS patients remains a challenge due to the lack of specific markers [103].

Dissecting interactions between OS cells and stroma may also provide insights into novel therapeutic targets. Evidence has been provided that exosomes originating from tumor cells induce a prometastatic inflammatory response by acting on MSC in the premetastatic niche at the primary tumor site [107,108]. Indeed, TGFβ on exosomes membranes induces the release of IL6 from MSCs and consequent activation of the oncogenic IL6/STAT3 signaling axis [107], independently of internalization of exosomes [109,110]. In turn, IL6/STAT3 signaling supports cancer cell proliferation, metastasis formation, tumor immunosuppression, and cancer stem cell self-renewal [111]. In OS patients, high IL6 serum levels seem to sustain activated STAT3 signaling, despite OS tumor cells expressing nearly undetectable levels of IL6, while presenting a TGFβ-induced prometastatic gene signature [107,112]. This effect is mediated by exosomes-bound TGFβ, confirming much higher levels of exosomes-associated TGFβ in OS patients compared with healthy control individuals [107]. Of clinical relevance, TGFβ mRNA expression negatively correlates with metastasis-free survival [107], while pharmacological inhibition of this prometastatic inflammatory loop reduces OS progression, pointing to IL6 and TGFβ inhibitors as novel attractive targets for the anti-cancer drug in combination with current chemotherapy [113,114].

miRNA deregulation in OS cells was reported since 2009 [115], and some miRNAs have been detected also in circulation [116]. Recently, a comprehensive miRNA screening of serum samples, collected from a cohort of OS patients, revealed that several relevant oncogenic miRNAs in OS, such as miR-21 and miR-214, were not detected as highly upregulated miRNAs in the circulation, while specific serum-based miRNA signatures associated with OS have been validated, including miR-17-5p and miR-25-3p [104]. Both miR-25-3p and miR-17-5p were enriched in exosomes and their expression has been confirmed to be deregulated in OS tissues [104]. Upregulated miR-25 levels in OS tissues promoted cell proliferation and tumor growth [117]. In addition, miR-25 has been reported as upregulated in osteo-differentiated MSCs too [118]. Of clinical relevance, serum miR-25-3p level at diagnosis was correlated with poor prognosis and reflected tumor burden, thus presenting a biomarker to monitor tumor growth and predict the prognosis of OS patients [104].

With the aim to design a predictive model to assess chemotherapy efficacy in OS, alterations of exosomal microRNAs and mRNAs content in serum of OS patients have been reported [119]. By profiling exosomes RNAs, OS patients with differential chemotherapeutic responses can be distinguished [119]. Twelve miRNAs were up-regulated and eighteen miRNAs were under-regulated in OS patients with poor chemotherapeutic response compared to responsive patients (*p* < 0.05) [119]. miR-124, miR133a, miR-199a-3p, and miR-385 were validated and significantly reduced in poor responder patients with an independent OS cohort, while miR-135b, miR-148a, miR-27a, and miR-9 were significantly over-expressed in serum exosomes [119]. Further, exosomal RNAs including Annexin2, Smad2, MTAP, CIP4, PEDF, WWOX, Cdc5L, P27 were differentially expressed depending on chemotherapeutic response [119]. Thus, evidence is provided for liquid biopsy application that exosomal miRNAs act as diagnostic biomarkers, while deregulation of exosomes RNA content is indicative of a poor chemotherapeutic response in OS patients [119].

In addition, a pilot study demonstrated dramatic transcriptomic alterations in serum exosomes RNA, by comparing metastatic and primary OS samples [120]. Potential driver mutations emerged in several genes, such as TP53, Axin1, FGFR, and FN1, and the cluster analysis indicated L1CAM, EGFR, PDGF, and growth pathway genes as the most relevant altered genes in the metastasis-related expression signature [120]. The prognostic value of the exosomes RNA-based expression signature was confirmed by using a cohort of 42 patients from public datasets [120]. In addition, the identified alterations in RNA profiling were fivefold greater in exosomes than in tissue, suggesting that circulating exosomes represent with efficacy the overall tumor burden [120]. By analyzing the profile of exosomal miRNAs, miR-675 is significantly increased in exosomes derived from metastatic OS cell lines and patients, but not in non-metastatic tumor cells [121]. MiR-675 modulates cancer cell proliferation, migration, and survival in several tumor types [122,123]. Coherently, exosomes of metastatic OS cells increase the migration and invasion of fibroblast cells in vitro, at least in part by acting on the miR-675 target CALN1 within recipient cells [121]. Finally, higher levels of serum exosomal miR-675 and lower levels of CALN1 in tumor tissues were associated with the metastatic phenotype in OS patients, pointing to circulating exosomal miR-675 as a valuable prognostic biomarker of lung metastasis and a therapeutic target [121].

Moreover, recent clinical data support the role of plasma exosomes-miR-101 as a circulating diagnostic biomarker for OS [124], as previously indicated for serum miR-101 level [125], providing insight into the potential use of exosomes-miR-101 within novel diagnostic and therapeutic strategies for the metastatic OS [124].

Among ncRNAs, circ0056285 and TRIM44 levels have been confirmed to be markedly up-regulated in serum exosomes of OS patients, with an opposite trend of miR-1244 level [126]. Circ0056285 in OS tissues was positively correlated with circ0056285 in serum exosomes, while miR-1244 and TRIM44 in OS tissues were not associated with their levels in serum exosomes [126]. Furthermore, the ROC curve confirmed that the level of exosomal hsa_circ0056285 had a high diagnostic value for OS [126]. Finally, due to its activity in mediating OS cell progression, TRIM44 could provide a new therapeutic target for OS [126].

A recent paper reports a higher level of PD-L1 in exosomes of a cohort of 70 OS patients, compared to healthy donors, with an AUC of 0.695 [102]. Of clinical relevance, levels of exosomal PD-L1 were higher in patients with pulmonary metastasis than in patients without metastasis [102]. PD-L1-associated poor prognosis may be due to immune suppression, chemotherapy resistance, and metastasis-related pathways [127]. The co-expression network of differentially expressed genes, with PD-L1 as the core gene, was related to cell–cell adhesion [102], and in accordance, the cadherin switch from E-cadherin to N-cadherin is a known key step of EMT, which occurs in metastasis progression. Of note, PD-L1 and N-cadherin levels in exosomes, and the ratio of N-cadherin/E-cadherin were proven to differentiate patients with metastasis and patients without metastasis, confirming high diagnostic sensitivity and specificity to predict the occurrence of lung metastasis in OS patients [102].

Finally, plasma exosomes-derived SENP1 protein may act as an independent prognostic predictor in OS patients [128]. Exosome-derived SENP1 levels in the patient’s plasma were related to tumor size, tumor location, necrosis rate, pulmonary metastasis, and surgical stage [128]. Both DFS and OS, at 1-year and 3-year, were worse in patients with higher plasma exosome-derived SENP1 levels compared with patients with lower plasma exosome-derived SENP1 levels [128].

Rhabdomyosarcoma (RMS) is thought to arise from primitive mesenchymal cells with myogenic differentiation [129]. RMS occurs as two main histologic subtypes: alveolar (ARMS) and embryonal (ERMS) histologies. The alveolar subtype is characterized by a chromosomal translocation t(2;13)(q35;q14), resulting in the fusion of the gene encoding the DNA binding domain of Paired Box 3 (PAX3) with the gene encoding the transcriptional activation domain of Forkhead Box O1 (FOXO1) [129]. Alternatively, the chromosomal translocation t(1;13)(p36;q14) results in a fusion between PAX7 on chromosome 1 and FOXO1, and occurs in a minor proportion of ARMS [130]. Clinically, the fusion oncoproteins dictate clinical tumor behavior and represent an independent negative prognostic marker [130]. Indeed, patients with fusion-positive ARMS present with advanced disease, and have high rates of tumor recurrence and poorer survival [131], despite current multimodality therapy. PAX3-FOXO1 acts as a transcriptional regulator and alters a number of genes involved in myogenic and developmental processes, proliferation, survival, migration, and metastasis [132], as well as several miRNA [133,134].

Exosomes have been demonstrated to be relevant mediators of paracrine effects of human RMS cells, in both fusion-positive and fusion-negative cell lines [135]. PAX3-FOXO1 directly modulates exosomes content of myoblasts, which resulted in pro-tumorigenic effects in recipient cells, and early metastasis of fusion protein-positive RMS [136], by acting on both miRNA, with networks centering on cancer and inflammation pathways, and proteins relevant in RMS tumor biology, including IGF1 and IGF1R, CDKN1B, SMAD 2/3, SIRT1, TP53, and the epigenetic regulator SMARCA [136]. Of note, miR-486-5p has been identified as a downstream effector in exosome-dependent oncogenic paracrine signaling, mediating increasing migration, invasion, and colony formation in recipient cell [136]. The analysis of serum samples from patients with RMS showed a tendency towards higher levels of miR-486-5p in exosomes, with a very high level in the one patient with fusion-positive alveolar RMS and a reduction upon chemotherapy and in the remission phase [136]. These results suggest the use of miR-486-5p as a potential serum exosome biomarker for fusion-positive RMS, to aid in diagnosis, assessment of response, and follow-up of patients. In addition, PAX-FOXO1 gene fusion transcript detection in cell-free RNA from blood exosomes has been assayed as a tumor-specific biomarker [137]. The analysis included 112 samples from 65 patients [137]. For patients with metastatic ARMS, 62% (*n* = 18) of initial liquid biopsies were positive, and 16 (89%) of them presented bone marrow metastases [137]. For all patients with primary localized ARMS, the liquid biopsy was negative at diagnosis [137]. In addition, results confirmed a correlation with the initial tumor status, since liquid biopsy was positive in 94% of patients with metastatic ARMS and initial metastatic involvement, whereas biopsies from all patients with localized tumors were negative [137]. However, a prospective validation is required for diagnostics and monitoring of soft-tissue sarcoma.

Liposarcoma is a common soft-tissue sarcoma subtype, and is subdivided into at least four different subtypes: (i) well-differentiated (WDLPS), (ii) de-differentiated (DDLPS), (iii) myxoid/round cell (MRC), and (iv) pleomorphic PLPS [138].

Chromosomal amplification at 12q13-q22, which contains the MDM2 and CDK4 genes, is the main hallmark of WDLPS and DDLPS, along with the frequent presence of genomic amplifications in 1p32, 1q21-24, and/or 6q23 and 13q-21-32. DDLPS recur as synchronous multifocal tumors, poorly responsive to therapy, and with the acquisition of metastatic capacity; thus, earlier therapeutic interventions are evidently needed [138,139]. In addition, no validated molecular biomarkers have been identified for prognosis, early detection of DDLPS progression or recurrence, or for drug resistance prediction [140].

RNA profiling of exosomes from the plasma of patients highlighted the role of circulating miRNAs as novel biomarkers for liposarcoma, and pinpointed their role in liposarcoma progression [141]. miR-25-3p and miR-92a-3p were showed to stimulate secretion of proinflammatory cytokine IL6 and TNF from tumor-associated macrophages by activating TLR7/8 receptors and NF-kB pathway [141]; IL6, in turn, promoted liposarcoma cell proliferation, invasion, and metastasis via interaction with the microenvironment [141]. Of note, the signature of circulating miRNAs may prove effective in a more accurate prognosis and in prediction of recurrence [141].

The great majority of DDLPS present a high level of MDM2, which alters the tumor suppressor function of wild-type TP53 [142]. Thus, the assessment of MDM2 through the FISH method is currently used in clinical practice for diagnosis of DDLPS [142]. Exosomes from DDLPS patients have been confirmed to contain significantly increased amounts of MDM2, which causes impaired p53 activity in recipient preadipocytes, release of matrix metalloproteinase 2 (MMP2), degradation of type IV collagen of basement membranes, with tumor invasion and metastasis; indeed, treatment with MDM2 inhibitors repressed these effects [142]. Last, collagen peptides generated by MMP2 can act as a chemoattractant for circulating tumor cells in the premetastatic niche [143]. Of note, therapeutic options based on targeting exosomal MDM2 show a good potential for treating DDLPS [142]. In particular, an ultrasensitive in situ hybridization (ISH) technique has been recently set to identify the MDM2 DNA in serum exosomes of liposarcoma patients, as a tool for diagnostic confirmation of specific DNA alterations, thereby facilitating tumor detection and diagnosis [144].

Lastly, in DDLPS, miRNAs of clinical relevance have also been identified, with high expression of miR-1246, -4454, and -619-5p both in serum and tumor tissues, with potential significance as biomarkers for early diagnosis or as therapeutic targets [145].

Synovial sarcoma (SS) is a high-grade STS that accounts for 10% to 20% of STSs, with high incidence of late metastases, most commonly to the lung, lymph nodes, and bone marrow [146]. The presence of the chromosomal translocation t(X;18)(p11.2;q11.2), which the SS18-SSX fusion gene originates from [147], is clinically useful as a diagnostic marker; however, it does not reflect disease progression [146]. To date, miRNA deregulation in SS tissues has been reported by several groups, with miR-17-5p18, miR-99b, miR-125a15, miR-18317 being upmodulated, and miR-14316 downmodulated [148,149,150]. Recently, a miRNA profiling analysis using SS patient serum pinpointed the potential clinical significance of miR-92b-3p for tumor monitoring [96]. Cell-free miR-92b-3p is stable and released within exosomes, and contribute to SS progression by mediating cell–cell communication [96]. miR-92b-3p is specifically overexpressed in primary brain tumors [151] and regulates the development of intermediate cortical progenitors [152], supporting the hypothesis of a neuroectodermal origin of SS [153]. Clinical relevance was validated in two independent cohorts, with serum miR-92b-3p levels significantly higher in SS patients in comparison to healthy individuals [96]. Moreover, serum miR-92b-3p discriminated patients with SS from the other STS patients and reflected tumor burden [96].

A recent study identified 199 common proteins in exomes secreted from SS cells, with the monocarboxylate transporter 1 (MCT1) as a novel surface marker, highly expressed in SS patient-derived exosomes compared with healthy individuals [63]. MCT1 has a key role in energy transfer by establishing a lactate shuttle system [154]. High MCT1 expression in several tumor cells is associated with oxidative metabolism [155], and silencing of MCT1 decreases resistance to chemotherapy in pancreatic adenocarcinoma cells [156], while it contributes to the inhibition of cellular proliferation, migration, and invasion of SS cells, indicating the therapeutic potential of MCT1 in SS [63]. Therefore, MCT1 may represent a novel therapeutic target. Circulating MCT1+CD9+ exosomes in serum reflected both tumor burden and treatment response in SS patients, with a significant correlation between MCT1 expression in tumors and prognosis [63], as confirmed in patients with breast cancer [154]. Overall, this work described a sensitive analytical technique for tumor monitoring, by detecting circulating exosomes of patients with SS [63].

Gastrointestinal stromal tumors (GIST) represent the most common mesenchymal tumor of the digestive tract and are thought to originate from the interstitial cells of Cajal (ICCs) or interstitial mesenchymal precursor stem cells [157]. GIST is frequently asymptomatic and often discovered in the advanced stage [158]. A great majority of GISTs contain oncogenic gain-of-function mutations in the receptor tyrosine kinase c-KIT (85%) or PDGFRA (3%) [158,159,160,161]. Small molecule tyrosine kinase inhibitors, most notably imatinib mesylate and sunitinib malate, were proven to be clinically effective in the advanced setting of inoperable or metastatic GIST [162]. Nevertheless, the median time to recurrence for patients receiving imatinib is only 2 years, with a median disease-specific survival of only 19 months with second- and third-line therapies, and the majority of patients eventually develop resistance [163].

Circulating levels of KIT-positive exosomes have been correlated with tumor burden and accumulated in plasma of patients with metastatic GIST as compared with primary disease; thus, quantitative changes in their levels might also indicate recurrence or metastasis [50]. Of relevance, a significant sorting of *p*-KITTyr719, total KIT, and SPRY4 has been confirmed in KIT-positive exosomes after treatment with imatinib of metastatic patients, indicative of response to therapy [50]. In accordance, a previous study identified the down-modulation of SPRY4A, FZD8, and PDE2A as markers associated with therapeutic response to imatinib in GIST biopsy specimens [164]. SPRY4 protein is a negative regulator of receptor tyrosine kinase-mediated signaling [165], suggesting that its internalization into exosomes is aimed to reduce the interference with downstream signaling [166]. SPRY4 levels are increased in metastatic GIST with respect to primary tumor and tumor tissue [50]. To assess whether these signatures can be used in a liquid-based assay to identify patients responsive to imatinib therapy, a larger cohort of clinical samples need to be analyzed [50].

Desmoplastic small round cell tumor (DSRCT) is a rare and aggressive mesenchymal tumor of adolescent and young adult males. This tumor primarily develops in the abdominal cavity from serosae surfaces, and metastasizes to the liver and lungs, with a poor prognosis [167]. The presence of the chromosomal translocation, t(11;22)(p13;q12), with the fusion between the EWSR1 gene on chromosome 22 and the WT1 gene on chromosome 1, is used for definitive diagnosis [168]. EWS–WT1 acts as a transcription factor and regulates several targets [169]. Recently, miRNAs enriched in circulating exosomes have been profiled in DSRCT patients, showing their function as potential indicators for disease status [170]. In total, 55 miRNAs have been confirmed to be significantly deregulated; among these, 14 miRNAs were highly modulated in at least one patient, and only five were expressed in all three patients, i.e., miR-34a-5p, miR-22-3p, miR-324-5p as upmodulated miRNAs, and miR-342-3p and miR-150-5p as downmodulated miRNAs [170]. These differentially expressed miRNAs resulted to be deregulated in several cancers and have a key role in modulating cell growth, proliferation, migration, and invasiveness [170]. Of note, miR-34a-5p has been implicated in promoting the multi-chemoresistance of OS [171,172]. Further, miRNAs upregulated in exosomes in all DSRCT patients have a tumor-suppressor function and may have an effect on the oncogenic potential of tumor cells [170]. The genes putatively targeted by upregulated miRNAs were involved in oncogenic signaling pathways, and included MAPK and RAS pathways, suggesting specific inhibitors as therapeutic strategies [170]. The limitations of the analysis are due to the few numbers of patients evaluated, the different stages of disease (diagnosis vs. progression), and the lack of different time points for the same patient [170].

A description of the advancements in technologies and methods available at present for exosomes assessment and purification from body fluids is beyond the objective of this review; however, it has been extensively analyzed in recent manuscripts, which present an overview of analytical platforms for detection and characterization of extracellular vesicles for translation in clinical practice [173,174]. The standardization of techniques has an obvious great impact for their use in routine practice. For this reason, in Table 1, we reported the isolation methods used for exosome detection in sarcoma patients, as evidence that some efforts in this direction have been made.

**Table 1:** Studies on application of exosome detection in sarcoma.

Tumor Type	Clinical or Research Application	*n* Patients	Exosomes Isolation Method	Potential Therapeutic Target	microRNAs/lncRNAs	Reference	Year
Desmoplastic small round cell tumor	Clinical, research: indicators of disease status	3	miRCURY™ Exosome isolation kit		miR-34a-5p, miR-22-3p, miR-324-5p, miR-150-5p, miR-342-3p	[170]	2019
Ewing Sarcoma	Clinical: diagnostic biomarker	6	Exosome isolation kit (Invitrogen)	EZH2		[80]	2016
Ewing Sarcoma	Research	30	Ultracentrifugation	HSAT2, HERV-K		[92]	2019
Ewing Sarcoma	Clinical: diagnostic and potentially prognostic	10	Immuno-pulldown (Dynabead)	CD99/MIC2, NGFR		[71]	2020
Ewing Sarcoma	Clinical (biomarker for diagnosis and tumor monitoring)	5	Qiagen Exo-RNeasy kit		46 mRNAs signature	[87]	2022
Fibrosarcoma	Clinical: diagnostic	10	Size exclusion chromatography	PCDH9	miR-1260b	[95]	2020
Gastrointestinal stromal tumor	Research	7	Ultracentrifugation	KIT		[175]	2014
Gastrointestinal stromal tumor	Clinical, research: drug resistance	4	Dynabeads M-450	SPRY4		[50]	2018
Liposarcoma	Clinical: potential prognostic	24	Ultracentrifugation	IL6, NFkB	miR-25-3p, miR-92a-3p	[141]	2017
Liposarcoma	Clinical: therapeutic option	16	ExoQuick (System Biosciences)	MDM2		[142]	2019
Liposarcoma	Clinical, research: biomarkers for early diagnosis or treatment targets in DDLPS	17	Ultracentrifugation		miR-1246, miR -4532, miR -4454, miR -619-5p, miR -6126	[145]	2021
OS	Clinical, research: therapeutic exploitation	18	Size-exclusion chromatography	TGFβ		[107]	2017
OS	Clinical: diagnostic and prognostic Marker	10	Ultracentrifugation		miR-25-3p, miR-17-5p	[104]	2017
OS	Clinical: chemotherapy sensitivity	93	Differential centrifugation		miR-124, miR133a, miR-199a-3p, miR-385	[119]	2017
OS	Clinical, research: biomarker of metastatic tumor	2	Sequential ultracentrifugation	CALN1	miR-675	[121]	2018
OS	Clinical, research: metastasis progression prediction	70	Density gradient centrifugation	PD-L1, N-cadherin, Rab27a		[102]	2020
OS	Clinical: therapeutic potential diagnostic potential	41	Differential centrifugation	BCL6	miR-101	[124]	2020
Rhabdomyosarcoma	Clinical, research: diagnosis, assessment of response, and follow-up	7	Sequential centrifugation and ExoQuick (System Biosciences)		miR-486-5p	[136]	2019
Synovial sarcoma	Clinical, research: monitoring tumor dynamics	9	Size exclusion chromatography		miR-92b-3p	[96]	2017
Synovial sarcoma	Clinical, research: therapeutic potential monitoring tumor burden and response to treatments	17	ExoScreen for profiling circulating exosomes	MCT1		[63]	2021

## 3. Preliminary Application of Liquid Biopsy in Sarcoma Patients

In this section, the most current reports in the literature, describing preliminary results on tissues from sarcoma, are reviewed as a proof-of-concept for further investigations in liquid biopsy, and pointing to potential use of other markers in novel diagnostic and therapeutic approaches. In ES, it has been reported that CD99neg exosomes contained a high level of miR-199a-3p, and mediated inhibition of AP-1 activity and expression of its target genes (i.e., MMP9, MMP1, and CCND1) in recipient cells [176]. In addition, the levels of miR-199a-3p in ES are decreased in metachronous metastases compared with primary tumors, modulating the expression of a key cell surface molecule in ES cells and reducing malignancy upon transfer to other tumor cells [176].

In a retrospective analysis of 45 tissue biopsy specimens from OS patients, the oncomiR miR-25-3p showed functional and clinical significance [177]; indeed, deregulated expression levels were significantly correlated with the presence of metastasis, and with poor overall survival and poor metastasis-free survival upon therapy [177]. Expression of Dickkopf WNT Signaling Pathway Inhibitor 3 (DKK3), a direct target of miR-25-3p, was inversely correlated with miR-25-3p in OS cells, confirming increased DKK3 levels due to miR-25-3p silencing [177].

Proliferation and apoptosis of OS cells has been confirmed to be regulated by exosome-mediated Hic-5 (hydrogen peroxide inducible clone 5, also known as TGFB1l1) [178]. Hic-5 was up-regulated in tumor tissues from OS patients [178,179,180], and upon release into exosomes, it affected the development of OS via activating Wnt/β-catenin signaling [178]. In accordance, high expression of β-catenin and some target genes have been reported to promote the development and metastasis of OS, especially lung metastasis [178].

Further, proliferation, migration, and invasion of OS cells are mediated also by exosomal miR-1307, which is highly expressed in human OS tissues and OS cell-derived exosomes, and inhibits the expression of the AGAP1 gene [181]. Clinical data suggest that the levels of miR-1307 and AGAP1 in OS tissues reflect the size of OS and the level of serum alkaline phosphatase (ALP), which may provide some value for the diagnosis and treatment of OS [181].

Recently, miR-101 detection in exosomes has been evaluated for the diagnosis of OS metastasis, and the therapeutic efficacy of exosome-mediated delivery of miR-101 has been confirmed [124]. miR-101 expression was markedly lower in metastatic compared to non-metastatic tissue specimens [124], confirming the tumor-suppressive function of miR-101 in OS invasiveness and metastasis [182]. Of note, miR-101 inhibited the homing of circulating tumor cells to the lung [124].

Plasma exosomes of OS patients have been analyzed in order to identify, among others, altered lncRNAs, and CASC15 has been confirmed to be significantly upregulated in OS exosomes compared with control, and coherently in tumor tissues with respect to normal tissues [183]. CASC5 is a lncRNA locus in chromosome 6p22 and has been reported to function as a tumor promoter in several tumors [183]. In vitro experiments verified that CASC15 promotes OS progression by targeting the miR-338-3p/RAB14 axis, and can act both as a biomarker and therapeutic target [183].

A recent study demonstrated that OS cells with high AXL expression promoted growth, invasion, and metastasis of tumor cells with low AXL expression through releasing linc00852-containing exosomes [184]. Receptor tyrosine kinase AXL and linc00852 have been identified to be significantly highly expressed in OS tissues and positively associated with metastasis and poor prognosis [185], and exosomal linc00852 has been proven to be a pivotal intercellular messenger in OS [184]. Increased expression of linc00852 caused AXL and AKT overexpression, thus promoting the progression of OS cells through the AXL-AKT pathway [184]. The AXL signaling is related to tumor cell self-renewal, invasion, metastasis, EMT, angiogenesis, and drug resistance [186]. Exosomal linc00852 constitutes a new potential tumor biomarker and a novel attractive molecular target for anti-neoplastic drugs in OS [184]. Further, exosomes represent an important mediator in the process of vascular remodeling and premetastatic niche generation [187], and can be applied as diagnostic markers, while exosome-based drug delivery systems are going to be improved [188].

Cross-talk between MSCs and OS has demonstrated the oncogenic potential of OS-exosomes also in recipient cells [189,190]. A recent study addressed the implications of OS-exosomes in the epigenetic reprogramming of MSCs [191]. As early indicators of transformation, OS-EV-treated MSCs and pre-osteoblasts showed higher expression of genes (MMP1, VEGF-A, ICAM1) related to bone microenvironment remodeling, and a significant upregulation of the intercellular adhesion molecule (ICAM1/CD54) [191]. Additionally, OS-EV-treated cells have been shown to acquire tumor-phenotype characteristics such as increased adhesion, proliferation, migration rate, and anchorage-independent growth [190]. In conclusion, MSCs may have transformed towards a cancer-associated fibroblast phenotype by the OS-exosomes treatment [191]. Thus, OS-exosomes dictated the fate of MSCs by modulating the epigenetic status, and also influenced the expression of genes related to bone microenvironment remodeling [191]. Further, OS-EV treatment provided a unique signature to the regulation of the expression of osteogenesis (RUNX2 and ALPL) and adipogenesis (PPARγ) related genes, with upregulated RUNX2 expression, and conversely downregulation of ALPL and PPARγ [191]. Of note, the more aggressive OS phenotypes often resemble early osteoprogenitors, while less aggressive tumors appear to share similarities with osteogenic MSCs that have progressed further along the differentiation cascade [191]. Probably, the response is primarily an immune reaction; however, it is not excluded that it is related to MSCs presenting a renewed phenotype [191].

In GIST, evidence has been provided that significant numbers of exosomes containing phosphorylated and not phosphorylated oncogenic KIT, as well as the exosomal markers CD9 and Annexin 1, are released by GIST cells and potently modulate surrounding stroma cells [175]. Indeed, upon uptake of GIST-derived exosomes, progenitor smooth muscle cells differentiate into tumor-promoting ICC-like cells [176]. Elevated expression of MMP1 is a prognostic factor for local recurrence and metastasis in human chondrosarcoma [192], and represents an attractive target for therapeutic strategies of metastatic tumors. However, clinical trials using MMP inhibitors in tumor therapy have not proven to be promising [193]. Accordingly, the continuous release of oncogene-containing exosomes from the tumor is likely to compensate for MMP inhibition, pointing to this exosome-mediated signaling as an alternative therapeutic approach to impair MMP-driven mechanisms of tumor invasion [175].

A comprehensive definition of the vesicular proteome profiling of highly purified GIST-derived exosomes identified a signature of previously unreported proteins, involved in tumor progression, angiogenesis, kinase signaling pathways, and immune regulatory components, pinpointing new diagnostic biomarkers and therapeutic targets [50]. Many of these proteins were validated using patient-derived KIT+ exosomes, and GIST tissues [47]. The proteome of exosomes consists of prognostic markers of GIST, including CDKN2A, EPHA4, FHL2, DPP4, EZR, HIF1A, and KCTD12, and proteins associated with imatinib response, such as SPRY4, SURF4, ALIX, and PDE2A [50]. The biological significance is evidenced by the oncogenic effect of exosomes on directly influencing cells in the tumor microenvironment. Additionally, the levels of exosomes and exosome-associated KIT and SPRY4 present a therapeutic significance [50].

## 4. Conclusions

At present, the use of liquid biopsy in sarcoma for tumor diagnosis, monitoring, and chemotherapeutic sensitivity has not yet been translated into clinical practice, due to limitations related, for instance, to the rarity of these tumors.

One of the major issues is the mesenchymal origin of these tumors, which poses a serious challenge in the isolation of CTCs [194]. In addition, the most recent ESMO recommendations on the use of circulating tumor DNA assays do not support the analysis of ctDNA for sarcoma [195]. The key role exerted by exosomes in tumorigenesis and progression of sarcoma has been confirmed in recent years, and the capability to detect exosomes in the plasma of cancer patients has been proven, unravelling potential roles in early diagnosis, prognosis prediction, tumor burden assessment, therapeutic responsiveness evaluation, and recurrence monitoring.

Even if methods for the isolation of exosomes have not yet been standardized for clinical practice, overall preliminary data collected in several subtypes of sarcoma and presented in our review clearly support the use of patient-derived exosomes as valuable tools for precision medicine, as validated for (i) diagnostic purposes, with potential application also in early stage tumors, (ii) tumor dynamics monitoring, and (iii) chemotherapeutic sensitivity prediction, in order to assess response to therapy and the follow up. In addition, a further translational application emerged for pharmacological purposes; indeed, engineering of EVs as nanocarriers of anticancer therapies is an active field offering new therapeutic opportunities to control primary tumors and metastatic disease [195]. Most studies have been performed on OS, Ewing Sarcoma, and liposarcoma, with few data on DSRCT, GIST, rhabdomyosarcoma, and synovial sarcoma. Of note, they all refer exclusively to circulating exosomes isolated from the serum or plasma of patients, and we do not include literature data on biomarkers supposed to be transported in the circulation into exosomes (e.g., miRNAs), if not otherwise explicitly proven. As a major limitation, at present, only a small patient cohort has been tested, thus highlighting the need for establishing collaborative projects between clinicians for these rare cancers.

Nevertheless, the clinical utility of EV detection in such a rare neoplasm is obviously emerging. As proof, in the PRIMMO clinical trial (registered as NCT03192059 in Clinicaltrials.gov, accessed on 10 May 2021) including patients with uterine sarcoma, a translational research package is included to evaluate immune response biomarkers in blood and tumor, including extracellular vesicles, in order to identify patients likely responsive to PD-1 inhibitors [196].

In conclusion, the implementation of clinical studies specifically designed to identify and monitor exosomes in liquid biopsy from patients diagnosed with sarcoma is thus expected in the near future. As mentioned, for use in clinical practice, a great effort should be directed in standardizing the methodology for the efficient isolation of exosomes from plasma or serum. Of note, with the rapid advancements of microfluidic chip development in liquid biopsy, it is expected that the interrogation of exosomes will become an informative tool for clinical application.

## Data Availability

Not applicable.
